# Dead Men Do Tell Tales

**Published:** 1914-10

**Authors:** Davina Waterson


### Abstracts and Selections.

### Dead Men Do Tell Tales.

BY DAVINA WATERSON.

Once upon a time, many a murderer was acquitted and many an innocent man hanged, because, to all appearances, they had or had not, been guilty of the crime.

But today an assassin has to reckon with the physicist, the chemist and the Rontgenologist, who start off on his track and force him to face damning, dispassionate scientific facts, but, with equal zeal bring these facts to clear a miserable man protesting his innocence, yet condemned beforehand by circumstantial evidence. Now, if a man is found walking quickly away from the place where a body has been found, and if the man has blood stains on him and on his pocketknife, naturally he is the murderer, so why spend a fee on an expert to prove the contrary?

Just such a case occurred in France. It was no use the man saying he had poached a rabbit, made a stew and burnt the skin and bones to avoid possible detection. He was condemned because the blood stains and a known hatred for the victim shouted guilty. Then came along a physicist and showed the blood to be that of a rabbit, for, by the unique methods of two professors, T. T. Reichert and A. P. Brown, it can be determined to what species of animal, bird or reptile the blood belongs, since every species has distinct crystallization. Experts claim to distinguish differences of nationality and it is no illogical optimism to state that their claim to prove consanguinity may prove to be correct. If Jacob had been able to set a scientist to work on Joseph's coat the brethren would have been confounded, and Reuben, the connivor at deliverance, extra triumphant. Ever since those days the malignant have tried to fix guilt on innocency by spattering the blood of animals on clothes or weapons, but it never can happen again in civilized countries.

A little while ago a mother murdered her little girl in a lonely spot, and "murder by person or persons unknown" was the verdict. A suspicious neighbor, who disliked the woman, took up the search privately, and one day found a blood-stained knife near the house. "Why, that's the knife I used to kill a rabbit last week. I put it there in the wall meaning to clean it," cried the mother.

Submitted to an expert, the knife told of human blood, blood shed a year ago, and the terrified mother confessed her guilt. It is now over seven years since an Italian physician, A. Lechanarzo, perfected the method of determining the age of a blood spot. Would Rizzio's blood, said to be renewed every year on the floor of Holyrood Palace, stand an investigation? I think the tourist, enjoying the induced thrill of horror, would rather the scientist kept out of the way!

Mutilation of a body is not always effectual, and has occasionally, by its very dexterity, convicted the real offender. A murderous butcher will, naturally, cut up his victim with precision, and a medical student or surgeon would do it in a skilful fashion. The mutilations by Jack the Ripper showed him to have considerable anatomical knowledge.

A physical defect, engendered by disease or habit, often guides an expert in detection, but when a man cuts his old father into 130 pieces and buries these separately in garden and field he naturally expects to lull suspicion, especially when he daily expresses surprise that the aged parent does not return from Paris.

Six months after the deed a farm hand dug up a hand, no clue apparently, except that a friend, a medico-legal expert, took note of certain callosities in the palm, rather peculiar ones, and soon after begged of the son his father's stick as a memento. The curiously carved knob exactly fitted the skeleton hand and the son was convicted of the murder. In the same way the body of a mutilated nun was identified by the callosities on the knees produced by constant kneeling; and Sir William Fergusson proved the identity of Livingstone (though it was hardly doubted) by showing an old ununited fracture in the left humerus. The structural deformations induced by occupation, often lead to the identification of a murdered man when he has been, say, a tailor, a barber or a shoemaker, while the condition of the teeth may show the victim to have been a printer or a potter, owing to the plumbism engendered.

Now, if a man is found shot through the head and with a pistol in his hand, what more rational than a verdict of suicide? But in real suicide the weapon is held so firmly that force is required to dislodge it. It seems as if the muscular spasm persists until rigor mortis occurs and sets it. Several experts have tried to make the hand of a corpse grip a weapon, but have never succeeded and their knowledge of this fact has often opened the avenue to detection of murder. Again, if you found your grandfather on the floor with a rope round his neck and the other end dangling from a nail in the wall, certainly you would say that he had hanged himself and his weight had broken the rope.

But the medico-legalist is as well up in knots as a sailor and knows a suicide would tie them one way, a murderer another. There was a case in Paris of a grandfather who had, apparently, hung himself in the manner described. But he had not tied the rope, declared the expert, and, in face of such uncanny knowledge, two neighbors confessed they had from their window seen the son-in-law strangle his father and arrange the other piece of rope.

A would-be murderer might advantageously study physiology, i. e., the physiological action of certain substances on the human body and might, also, if he intends hiding the corpse, read concerning flora and fauna in the dead. The advice of Moquin-Tandon, some time professor of natural history in Paris University, was often asked by legal doctors because he had made a special study of the latter. The body of a little girl was found tightly packed up in a soap box, and the mother, when found in another city, tried to fix the crime on a friend and as happening two months ago. It was then the end of July. By a careful study of the flies and larvae found in the remains, the expert proved the body to have been in the box since the preceding February, and the mother confessed to having killed the child on February 27. In the same way the examination of the larvae on the body of a child hidden up a chimney proved death to have taken place fully two years previously, and this verdict led to the acquittal of a suspected person. There are certain fauna which begin their work soon after death, then disappear to give place to others, but the succession is invariable and marks time for the scientist.

The different physiologic action of fire on a dead body and a living one was not known by the man who rushed frantically to his neighbors, saying he had found his wife lying across a chair by the fire badly burned from waist to knees and also on the neck. Unfortunately for him the doctor pointed out that burns made before death contained serum and there was no vesication (thin serous fluid under the skin), moreover, the fire could not have passed from waist to throat. The man then confessed to strangling his wife and afterwards setting fire to her.

The student who murdered his aged uncle by drowning had clearly not taken chemistry in his studies or he would not have been so confounded when brought to justice. He had wound nine yards of thick lead tubing round the body to sink it. Surely enough? But a little knowledge of chemistry would have made him make a few incisions for the escape of the natural gases, as these brought up the corpse in spite of the heavy weight.

Lynx-eyed science is rendering it ever more difficult to dispose of a body or hide the crime of murder. Human blood and hair and bones have characteristics distinctly their own. The "gory knife" of melodrama is no longer sufficient to fix a crime, and even if, as seems possible, the penny novelettist should kill his hero with radium, why, the physicist would come along with the electroscope and with it absolutely refute or confirm the accusation.—*Alienist and Neurologist.*

